# Differentially expressed genes in the head of the 2^nd^ instar pre-molting larvae of the *nm2* mutant of the silkworm, *Bombyx mori*

**DOI:** 10.1371/journal.pone.0180160

**Published:** 2017-07-20

**Authors:** Pingyang Wang, Simin Bi, Fan Wu, Pingzhen Xu, Xingjia Shen, Qiaoling Zhao

**Affiliations:** 1 School of Biotechnology, Jiangsu University of Science and Technology, Zhenjiang Jiangsu, China; 2 The Sericulture Research Institute, Chinese Academy of Agricultural Sciences, Zhenjiang Jiangsu, China; 3 Industrial Crops Institute, Hubei Academy of Agricultural Sciences, Wuhan, China; Kunming University of Science and Technology, CHINA

## Abstract

Molting is an important physiological process in the larval stage of *Bombyx mori* and is controlled by various hormones and peptides. The silkworm mutant that exhibits the phenotype of non-molting in the 2^nd^ instar (*nm2*) is incapable of molting in the 2^nd^ instar and dies after seven or more days. The ecdysone titer in the *nm2* mutant is lower than that in the wildtype, and the mutant can be rescued by feeding with 20E and cholesterol. The results of positional cloning indicated that structural alteration of *BmCPG10* is responsible for the phenotype of the *nm2* mutant. To explore the possible relationship between *BmCPG10* and the ecdysone titer as well as the genes affected by *BmCPG10*, digital gene expression (DGE) profile analysis was conducted in the *nm2* mutant, with the wildtype strain C603 serving as the control. The results revealed 1727 differentially expressed genes, among which 651 genes were upregulated and 1076 were downregulated in *nm2*. BLASTGO analysis showed that these differentially expressed genes were involved in various biological processes, cellular components and molecular functions. KEGG analysis indicated an enrichment of these differentially expressed genes in 240 pathways, including metabolic pathways, pancreatic secretion, protein digestion and absorption, fat digestion and absorption and glycerolipid metabolism. To verify the accuracy of the DGE results, quantitative reverse transcription PCR (qRT-PCR) was performed, focusing on key genes in several related pathways, and the results were highly consistent with the DGE results. Our findings indicated significant differences in cuticular protein genes, ecdysone biosynthesis genes and ecdysone-related nuclear receptors genes, but no significant difference in juvenile hormone and chitin biosynthesis genes was detected. Our research findings lay the foundation for further research on the formation mechanism of the *nm2* mutant.

## Introduction

The silkworm, *Bombyx mori*, a holometabolous lepidopteran, is an experimental model for molecular entomology in the fields of genetics, physiology and biochemistry [1]. The insect epidermis lacks elasticity; as a result, insects must molt several times during the larval period to allow insect growth and development. Molting is an important physiological process in the silkworm and is controlled by prothoracicotropic hormone (PTTH), 20-hydroxyecdysone (20E), juvenile hormone (JH) and neuropeptides. When it becomes necessary to molt, ecdysone is synthesized in the prothoracic gland promoted by PTTH, after which activated 20E is combined with the heterodimeric nuclear receptor EcR-USP to promote the expression of early genes. Early-late genes are then activated by the early genes, followed by a series of late genes activated by the early-late genes, such as eclosion hormone biosynthesis genes, neuropeptide genes, cuticular protein genes and hydrolase genes, and the activating signals are amplified by the key nuclear receptor βFTZ-F1 [2]. When the 20E titer decreases in the late phase of molting, numerous pigmentation-related enzymes are expressed and transported to the epidermis. The resulting pigments participate in the formation of a new epidermis [3, 4]. Molting is mainly controlled by molting hormone and juvenile hormone. However, the pupating process requires the disappearance of juvenile hormone, and ecdysone plays a dominant role [5, 6].

During molting, cuticular proteins play key roles. A large number of cuticular proteins are synthesized during the formation of a new epidermis, and the same proteins are degraded in the apolysis of the old epidermis. It has been demonstrated that more than 1% of the genes are cuticular protein genes (CPGs) in almost all insect genomes [7]. In the *Bombyx mori* genome, more than 200 CPGs are predicted [7, 8] and can be classified into 12 families based on the characteristics of conservative motifs [9]. Cuticular proteins act mainly as structural proteins of the epidermis [10]. Additionally, they play roles in other processes, such as locomotion, feeding, stress resistance and immunity [11–14]. Thus, CPGs play irreplaceable roles in insect development and reproduction.

Studying the functions of molting-related genes using mutants is a good way to analyze the mechanisms leading to molting. A 2^nd^ instar non-molting (*nm2*) mutant that we discovered in the silkworm variety C603 provides a good model for studying the mechanisms underlying molting. The *nm2* mutant appears normal in the first instar, but at the beginning of the pre-molting stage of the second instar, it is unable to molt and becomes lustrous, which lasts for 6 to 8 days, followed by death of the larva. Genetic and clone mapping analyses revealed that a deletion of 217 bp in the open reading frame of the cuticular protein gene *BmCPG10* is responsible for the phenotype of the *nm2* mutant [15]. In this study, the head of the *nm2* mutant in the pre-molting stage was subjected to digital gene expression (DGE) profile analysis, and the wildtype C603 served as the control. The differentially expressed genes according to DGE analysis and the key genes in ecdysone, juvenile hormone and chitin biosynthesis, cuticular protein genes and ecdysone-induced nuclear receptor genes were analyzed and verified using quantitative reverse transcription polymerase chain reaction (qRT-PCR). The possible mechanism underlying the development of the *nm2* mutant phenotype is explained based on the results of our DGE analysis and previous studies.

## Materials and methods

### Silkworm rearing and tissue isolation

The *nm2* mutant and wildtype C603 silkworm strains were supplied by the Sericulture Research Institute, Chinese Academy of Agricultural Sciences (Zhenjiang, China). The larvae were fed fresh mulberry leaves under standard conditions: 25 ±2°C temperature, 12-h light/12-h dark photoperiod and 65 ±5% relative humidity. The 2^nd^ instar pre-molting larvae of the wildtype and the *nm2* mutant were dissected and subsequently stored at −80°C.

### RNA extraction

Total RNA was extracted from the head of the 2^nd^ instar pre-molting larvae using RNAiso Plus (TaKaRa, China), according to the instructions of the manufacturer. After treatment with DNase I (TaKaRa, China) to degrade DNA contamination, the concentration of total RNA was determined using a NANODROP1000 microspectrophotometer (Thermo, USA). Total RNA quality was determined based on the 260/280 absorbance ratio and electrophoresis and then stored at −80°C.

### Digital gene expression profile

The treated mRNA was enriched using oligo (dT) magnetic beads. By mixing with fragmentation buffer, the mRNA was fragmented into short fragments. Then, the first strand of cDNA was synthesized using random hexamer primers. Buffer, dNTPs, RNase H and DNA polymerase I were subsequently added to synthesize the second strand. Next, the double-stranded cDNA was purified with magnetic beads. End repair and 3’-end single A (adenine) nucleotide addition were then performed. Finally, sequencing adaptors were ligated to the fragments, and the fragments were enriched via PCR amplification. During the quality control (QC) step, an Agilent 2100 Bioanaylzer and an ABI StepOnePlus Real-Time PCR System were used to qualify and quantify the sample library. The library products were then ready for sequencing via the Illumina HiSeqTM 2000 platform at the Beijing Genomics Institute (BGI).

### DGE data analysis

The primary sequencing data produced by the Illumina HiSeqTM 2000 platform, referred to as raw reads, were subjected to quality control (QC) procedures to determine whether a resequencing step was needed. After QC, the raw reads were filtered into clean reads, which were aligned to reference sequences from the silkworm genome database, SilkDB (http://silkworm.swu.edu.cn/silkdb/). QC of the alignment was performed to determine whether resequencing was needed. The alignment data were utilized to calculate the read distribution and mapping ratio based on the reference genes. If the alignment results passed QC, downstream analyses, including gene expression and deep analyses, proceeded based on the observed gene expression (e.g., principal component analysis/correlation/screening of differentially expressed genes). Deep analyses based on DGE were also performed, including Gene Ontology (GO) enrichment analysis and KEGG pathway enrichment analysis.

RSEM (RNA-Seq by Expectation Maximization) [16] is a quantification tool that computes maximum likelihood abundance estimates using the expectation maximization (EM) algorithm in its statistical model, including the modeling of paired-end (PE) and variable-length reads, fragment length distributions, and quality scores, to determine which transcripts are isoforms of the same gene. Expression levels were calculated according to the FPKM (Fragments Per Kb per Million fragments) method. The differentially expressed genes were filtered by referring to the digital gene expression profiles reported by Audic et al. [17]. P-values corresponding to differential gene expression were calibrated via multiple hypothesis testing, and the P-value threshold was determined by controlling the FDR (false discovery rate) [18]. The significance of gene expression differences was judged using |Log_2_ (*nm2*/wildtype)| ≥ 1 and an FDR ≤ 0.001 as the default thresholds.

### Quantitative reverse transcriptase PCR

The heads of *nm2* mutant and wildtype C603 larvae in the pre-molting stage were collected. Total RNA was extracted using RNAiso Plus (TaKaRa, China), according to the instructions of the manufacturer. After treatment with DNase I (TaKaRa, China), cDNA was synthesized using Reverse Transcriptase M-MLV (RNase H-) kit (TaKaRa, China), according to the instructions of the manufacturer, and was then diluted to 50 ng/μL to serve as the template for qRT-PCR. The 20-μL reaction system included 1 μL of primers (10 μmol/L, S1 Table), 1 μL of cDNA, 10 μL of 2×SYBR^®^ Premix Ex Taq™ (Tli RNaseH Plus) (TaKaRa, China) and 8 μL of ddH_2_O. After transient centrifugation, qRT-PCR was performed using a LightCycle 96 Real-time PCR System (Roche, Switzerland) with the following reaction program: three-step reaction protocol consisting of 45 cycles of 95°C for 10 s, 58°C for 10 s and 72°C for 10 s, after a 10-min step of pre-degeneration, followed by melting. The quality of the qRT-PCR product was tested through melting curve analysis, and relative expression was calculated using the 2^–ΔΔCt^ method [19], with the average of three house-keeping genes, *RPL3* (*BGIBMGA013567*), *GAPDH* (*BGIBMGA007490*) and *BmActin3* (*BGIBMGA005576*), serving as the reference. The qRT-PCR results were compared with the DGE results to verify the accuracy of the DGE data.

## Results

### Analysis of DGE libraries

From the DGE libraries, we acquired 12,912,619 and 12,900,347 clean reads from the 13,129,213 and 13,129,324 raw reads after sifting of 216,594 and 216,594 adapter reads, 21,750 and 23,019 reads in which unknown bases were more than 10% and some low quality reads that the percentage of low quality bases was over 50% in a read in the wildtype C603 and the *nm2* mutant, respectively. The ratio of clean reads to raw reads was 98.35% in the wildtype and 98.26% in the *nm2* mutant (Table 1). The GC% for *nm2* (50.88%) was slightly higher than that for the wildtype (49.65%). The clean reads from the two libraries contained unknown N bases but at proportions as low as 0.0034% and 0.0036%, respectively (Table 1).

**Table 1:** General information on reads from the two libraries.

Category	Parameter	Wildtype	*nm2* mutant
Raw data	Total Reads	13,129,213	13,129,324
	Total bases	643,331,437	643,336,876
	Adapter reads (%)	216,594 (1.65)	228,977 (1.74)
	Number of N (%)	21,750 (0.0034)	23,019 (0.0036)
	GC (%)	49.67	50.89
Clean data	Total Reads	12,912,619	12,900,347
	Total bases	632,718,331	632,117,003
	Number of N (%)	21,334 (0.0034)	22,593 (0.0036)
	GC (%)	49.65	50.88
	Clean data/Raw data (%)	98.35	98.26
Quality Control	Clean Read Q20(%) ≥ 95 (Y or N)	98.7 (Y)	99.1 (Y)
	Clean Read Q30(%) ≥ 90 (Y or N)	95.7 (Y)	97.0 (Y)
	Clean Reads ≥ 10M (Y or N)	12.91M (Y)	12.90M (Y)
	Gene Unique Mapping Ratio(%) ≥ 80 (Y or N)	91.58 (Y)	88.62 (Y)
	Genome Mapping Ratio(%) ≥ 50 (Y or N)	81.61 (Y)	81.13 (Y)
Gene-mapping	Total Mapped Reads (%)	7,163,898 (55.48)	7,755,547 (60.12)
	Perfect Match (%)	4,992,541 (38.66)	5,492,892 (42.58)
	Mismatch (%)	2,171,357 (16.82)	2,262,655 (17.54)
	Unique Match (%)	6,561,285 (50.81)	6,873,863 (53.28)
	Multi-position Match (%)	602,613 (4.67)	881,684 (6.83)
	Total Unmapped Reads (%)	5,748,720 (44.52)	5,144,799 (39.88)
Genome-Mapping	Total Mapped Reads (%)	10,538,011 (81.61)	10,465,526 (81.13)
	Perfect Match (%)	7,374,523 (57.11)	7,435,737 (57.64)
	Mismatch (%)	3,163,488 (24.5)	3,029,789 (23.49)
	Unique Match (%)	8,452,626 (65.46)	8,328,786 (64.56)
	Multi-position Match (%)	2,085,385 (16.15)	2,136,740 (16.56)
	Total Unmapped Reads (%)	2,374,608 (18.39)	2,434,821 (18.87)

**Y:** Meaning sample passed this QC item.

**N:** Meaning sample failed of passing this QC item.

**M:** representing the logogram of “MEGA”.

**Fig 1:**
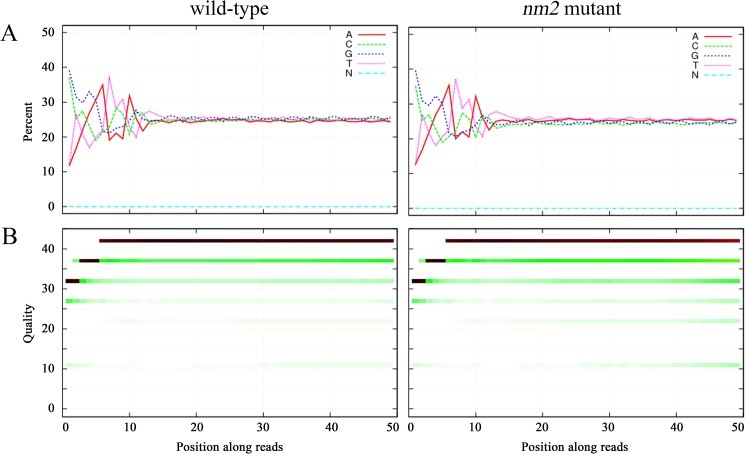
Analysis of read data. (A) Percentage composition of bases in the reads. The four types of base and unknown bases (N) were uniform from the 10^th^ base. (B) Distribution of quality. High quality was observed from the 6^th^ base to the last and even for the first 6 bases.

Evaluation of the reads showed that the base distribution of the reads in the two libraries was decentralized in the first 10 bases, which was normal because of the sequencing adaptors. Thereafter, the four type of bases and unknown N bases became uniform, thus meeting the requirements for follow-up analysis (Fig 1A). The analysis of the quality distribution showed a high quality for bases from the 6^th^ to the last base and even for the first 6 bases (Fig 1B). In addition, the results of Q20 and Q30 analysis all met the requirements for follow-up analysis (Table 1).

### Read mapping analysis

By aligning the clean reads to the reference genome database SilkDB (http://silkworm.genomics.org.cn/), we found that 81.61% and 81.13% of the clean reads could be aligned to the genome in the wildtype and the *nm2* mutant, respectively, among which 57.11% and 57.64% were completely aligned to the genome, and 24.5% and 23.49% were partially aligned to genome. In addition, 65.46% and 64.56% of the clean reads could be mapped to unique sequences, while 16.15% and 16.56% mapped to sequences at multiple positions in the wildtype and the *nm2* mutant, respectively. The clean reads were also aligned to 14623 known genes [20], and the percentages of total mapped reads, perfectly mapped reads, partially mapped reads, uniquely mapped reads and multiply mapped reads in the wildtype were 55.48%, 38.66%, 16.82%, 50.81% and 4.67%, respectively, but in the *nm2* mutant, the corresponding values were 60.12%, 42.58%, 17.54%, 53.28% and 6.83% (Table 1). Quality assessments, including the unique gene mapping ratio and the genome mapping ratio, also met the requirements for follow-up analysis (Table 1).

**Fig 2:**
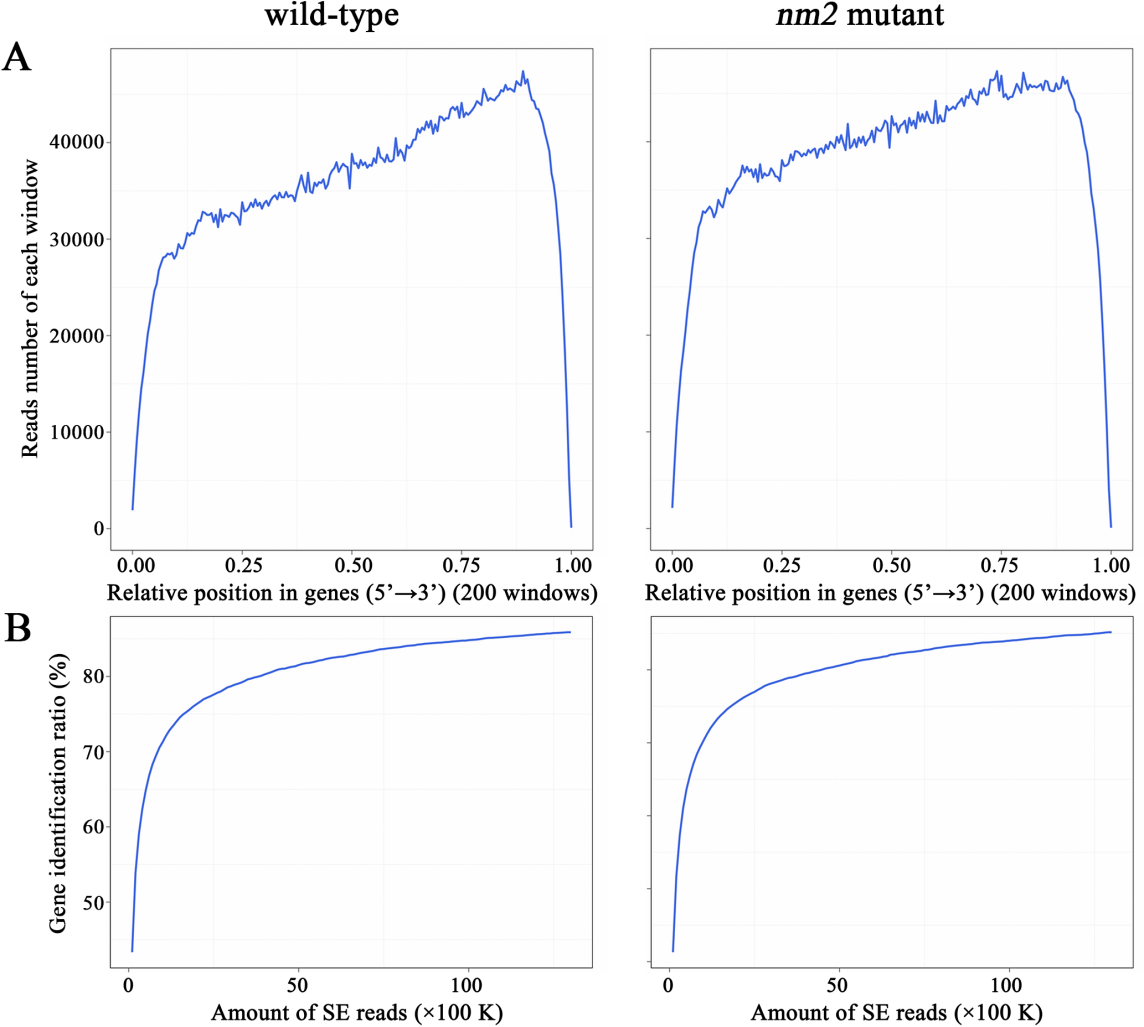
Analysis of mapping data. (A) Reads random from the wildtype and the *nm2* mutant. Most of the clean reads could be mapped to the middle of genes. (B) Sequence saturation of the wildtype and the *nm2* mutant. The saturation increased almost linearly with the increase in the number of reads in the range of 0 to 70%.

Reads random analysis showed that most of the clean reads could be mapped to the middle of genes and fewer to the 5 'and 3' ends (Fig 2A). Sequence saturation analysis showed that in the range of 0 to 70%, saturation increased in nearly linearly with an increase in the number of reads. Only approximately 1 Mega reads reached a saturation of 75%, while the clean reads obtained from DGE analysis reached 13 Mega, and the sequencing depth met the requirements for follow-up analysis (Fig 2B).

### Analysis of differentially expressed genes

In the two libraries, 11,856 genes were found to be expressed in the wildtype (81.08% of all 14,623 genes) and 11,751 in the *nm2* mutant (80.36%), among which 11,231 genes were expressed in both the wildtype and the *nm2* mutant. In addition, 625 genes were expressed specifically in the wildtype, whereas 520 were expressed specifically in the *nm2* mutant (S2 Table).

**Fig 3:**
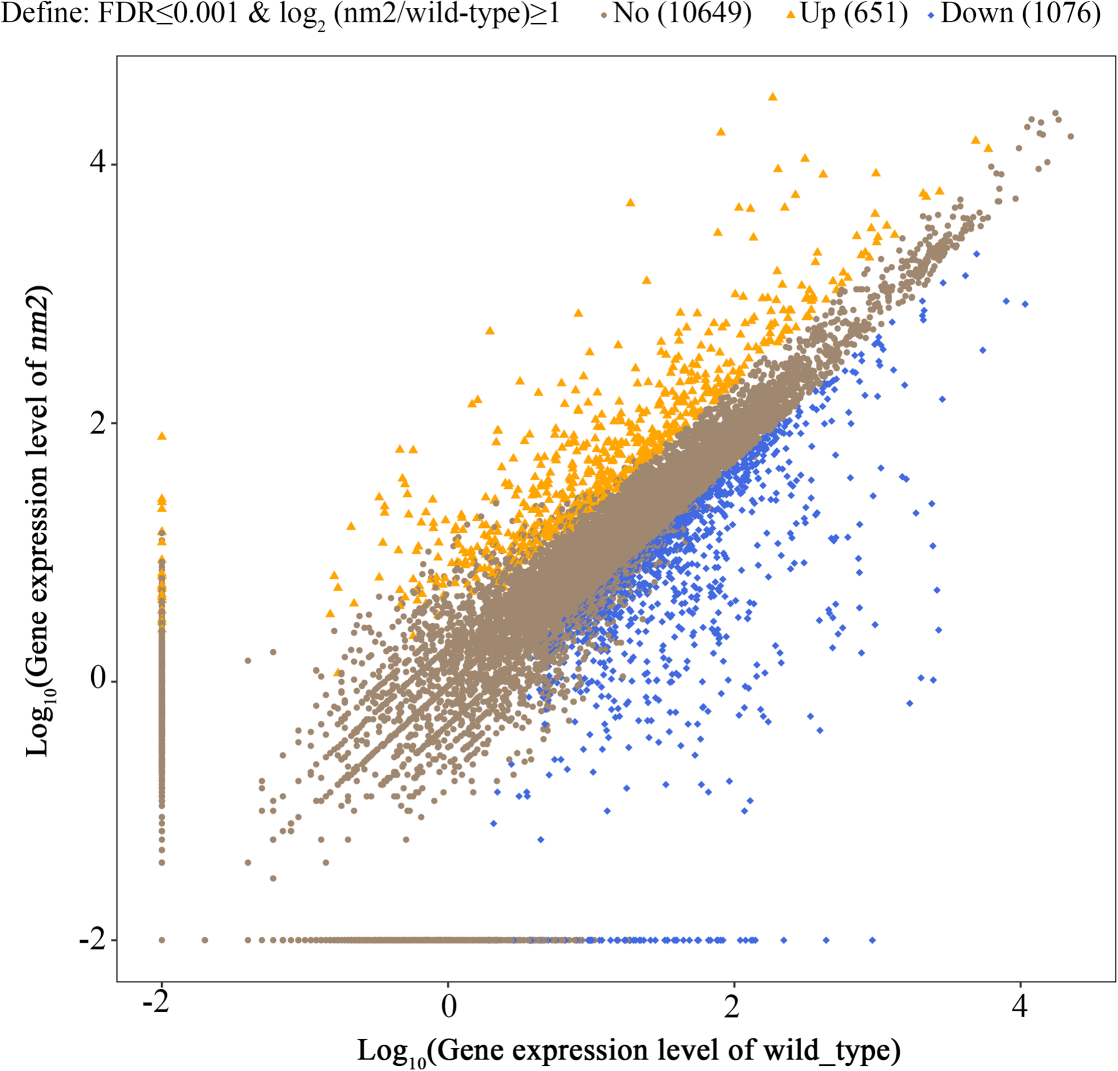
Scatter plots of all expressed genes in each pairwise. Blue blots mean down-regulation genes, orange blots mean up-regulation genes and brown blots mean non-regulation genes. The screening threshold was on top legend.

The scatter plots presented the distribution of differentially expressed genes in screening threshold dimensions (Fig 3). We observed 651 upregulated genes in the *nm2* mutant and 1076 downregulated genes, according to the default thresholds of an FDR≤0.001 and an FPKM ratio = |log_2_ (*nm2*/wildtype)| ≥1 (S3 Table).

**Fig 4:**
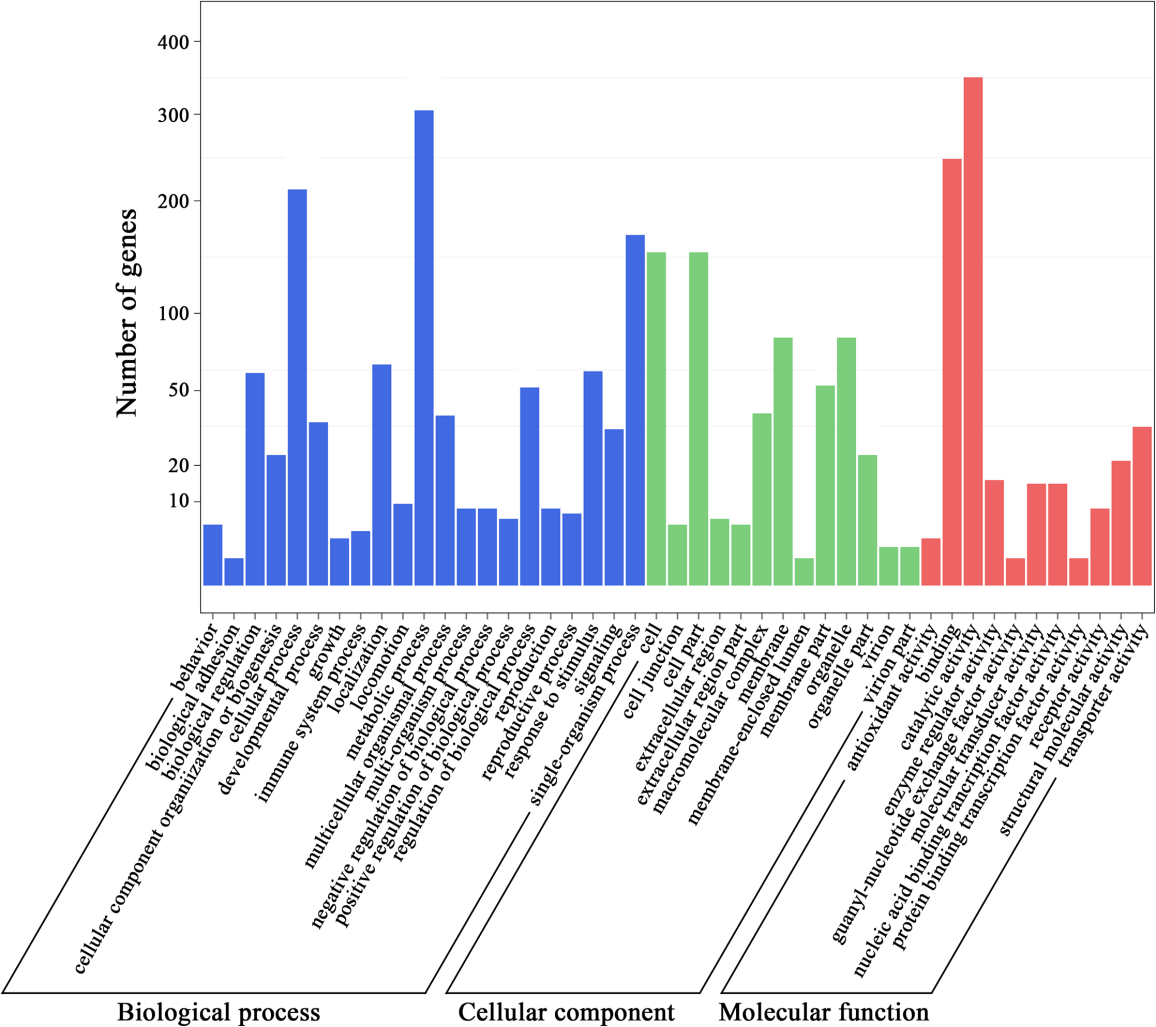
GO enrichment of biological processes, cellular components and molecular functions. The differentially expressed genes were mainly related to biological processes such as metabolic processes, cellular processes, single-organism processes, localization, the response to stimulus and biological regulation; cellular components such as the cell, cell parts, membranes and organelles; and molecular functions such as catalytic activity, binding and transporter activity.

The GO enrichment analysis showed that these 1727 differentially expressed genes (651 upregulated genes and 1076 downregulated genes) were mainly involved in biological processes such as metabolic processes, cellular processes, single-organism processes, localization, the response to stimulus and biological regulation; cellular components such as the cell, cell parts, membranes and organelles; and molecular functions such as catalytic activity, binding and transporter activity (Fig 4, S4 Table).

**Fig 5:**
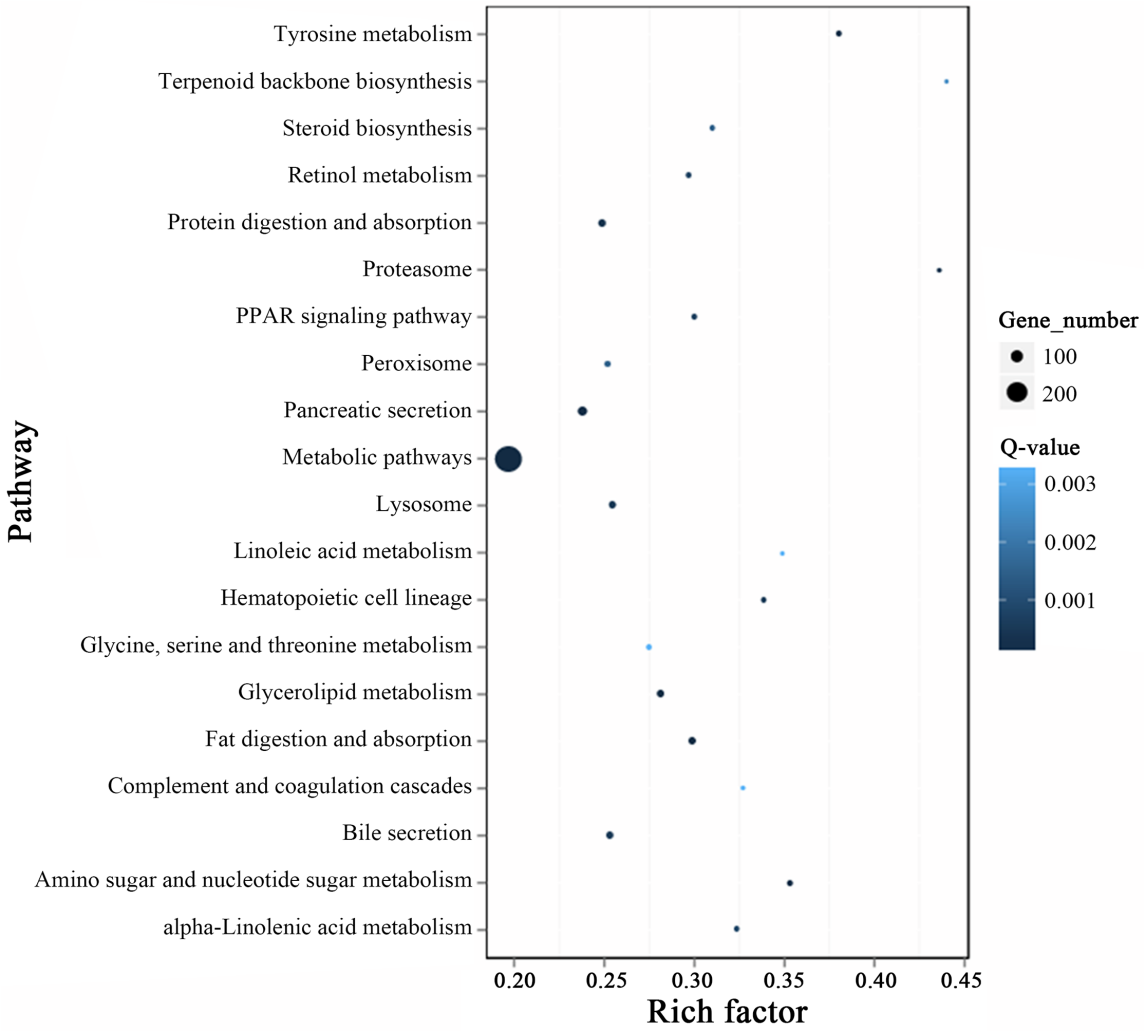
Statistics of top 20 pathway enrichment of differentially expressed genes in each pairwise. Rich factor is the ratio of differentially expressed gene numbers annotated in this pathway term to all gene numbers annotated in this pathway term. Greater rich factor means greater intensiveness. Q-value is corrected P-value ranging from 0~1, and less Q-value means greater intensiveness.

KEGG cluster analysis was performed for these 1727 differentially expressed genes using the KEGG database (http://www.genome.jp/kegg/kegg2). Approximately 1130 genes were mapped to 240 pathways, among which 273 genes (159 upregulated and 114 downregulated) were mapped to the first of the top 5 pathway categories: metabolic pathways. The other categories in the top 5 were pancreatic secretion (42 upregulated genes among 68 total genes), protein digestion and absorption (28 upregulated genes among 49 total genes), fat digestion and absorption (26 upregulated genes among 46 total genes) and glycerolipid metabolism (27 upregulated genes among 45 total genes) (Fig 5, S4 Table).

### Verifying the accuracy of the DGE data

To verify the accuracy of the DGE data, we analyzed the expression of several housekeeping genes as well as several target genes that had been investigated in previous studies. Additionally, some key genes, including partial differentially expressed genes based on DGE, ecdysone, juvenile hormone and chitin biosynthesis and metabolism genes, and several nuclear receptor genes, were verified using qRT-PCR (Table 2, S1 Table).

**Table 2:** Comparison of FPKM and qRT-PCR results.

Team	Gene id.	Description	Ratio (*nm2*/wildtype)		consistency	
			FPKM	qRT-PCR	Up	Down
(A)	BGIBMGA005576	actin3	1.17	-	-	-
	BGIBMGA007490	glyceraldehyde-3-phosphate dehydrogenase	0.85	-	-	-
	BGIBMGA013567	ribosomal protein L3	0.90	-	-	-
(B)^*^	BGIBMGA002602	Insect cuticle protein	2.37	3.56	√	
	BGIBMGA002602	Insect cuticle protein	2.37	0.00	-	-
	BGIBMGA009184	putative peptidase	86.16	586.77	√	
(C)	BGIBMGA000158^**^^,^ ^#^	Glucose-methanol-choline oxidoreductase	7.27	0.98	×	
	BGIBMGA002548	Insect cuticle protein	266.56	2.49	√	
	BGIBMGA004000	Glycoside hydrolase	0.03	0.08		√
	BGIBMGA005277	Insect cuticle protein	177.86	6.66	√	
	BGIBMGA006066^**^^,^ ^#^	Glycosyl hydrolase	21.58	0.86	×	
	BGIBMGA007888	Steroid hormone receptor	0.01	0.05		√
	BGIBMGA009467 ^#^	Glucosamine-6-phosphate isomerase	0.11	0.59		√
	BGIBMGA009688	Steroid hormone receptor	0.01	0.03		√
	BGIBMGA009743	Peptidase	0.00	0.45		√
	BGIBMGA010240	Glycoside hydrolase	0.01	0.06		√
	BGIBMGA010811	Glycoside hydrolase	7.36	3.79	√	
	BGIBMGA010812	Glycoside hydrolase	5.48	3.09	√	
	BGIBMGA013002	Glucose-methanol-choline oxidoreductase	0.03	0.46		√
	BGIBMGA013009	Glucose-methanol-choline oxidoreductase	0.01	0.12		√
	BGIBMGA013237 ^#^	Cytochrome P450	16.01	1.11	√	
(D)	BGIBMGA000368	dib	0.74	1.11		×
	BGIBMGA001678	Neverland	1.94	11.64	√	
	BGIBMGA001753	spook	2.46	2.84	√	
	BGIBMGA005496^**^	Sad	0.47	5.47	√	
	BGIBMGA006916	cyp18a1	0.23	0.43		√
	BGIBMGA006936	Phm	0.97	1.06		×
	BGIBMGA010239	cyp314a1	0.75	0.05		√
(E)	BGIBMGA000772	JH epoxide hydrolase (JHEH)	0.93	0.65		√
	BGIBMGA008815^**^	JH diol kinase (JHDK)	2.21	0.68	×	
	BGIBMGA010392	JH acid methyltransferase (JHAMT)	1.22	1.08	√	
	BGIBMGA013930	JH esterase (JHE)	1.43	0.84	×	
(F)	BGIBMGA001609	UDP-N-aeetylglueosamine pyrophosphorylase	2.59	1.33	√	
	BGIBMGA004221	Glucose-6-phosphate isomerase	0.86	1.09		×
	BGIBMGA007517	Glutamine: fructose-6-phosphate-aminotransferase	1.47	1.26	√	
	BGIBMGA011646	β-N-acetyl-glucosaminidase	0.40	0.63		√
(G)	BGIBMGA000716	FTZ-F1	6.08	5.04	√	
	BGIBMGA006183	USP	1.80	1.45	√	
	BGIBMGA006767	ecdysone receptor	0.73	0.69		√
	BGIBMGA006839	E75	0.2	0.55		√
	BGIBMGA007888	HR4	0.01	0.05		√
	BGIBMGA009688	HR3	0.01	0.03		√
	BGIBMGA002964	HR38	1.28	0.71	×	
	BGIBMGA007914^**^	HR39	1.51	0.36		√
(H)^***^	-	glutathione S-transferase	-	1.36	-	-
	-	glutathione S-transferase	-	1.31	-	-

(A) The reference genes; (B) the two main genes identified in previous research; (C) partial differentially expressed genes based on DGE; (D) ecdysone biosynthesis and metabolism genes; (E) juvenile hormone biosynthesis and metabolism genes; (F) chitin biosynthesis and metabolism genes; (G) several nuclear receptor genes; (H) *nobo*

*: Presenting one gene, BmCPG10 [15]. Two results from qRT-PCR based on two primers are located at different sites.

**: The results for these genes were contradictory between DGE and qRT-PCR.

***: Presenting one gene, glutathione S-transferase (*nobo*), based on two primers.

#: Representing for specific genes that were without significant differences in team (C).

The fold-changes of three housekeeping genes, *BmActin3* (Actin), *GAPDH* (glyceraldehyde-3-phosphate dehydrogenase) and *RPL3* (ribosomal protein L3), between the wildtype and the *nm2* mutant were approximately equal to one, which preliminarily verified the accuracy of the DGE data. *BmCPG10* [15] and *BmCP-like* [21] were upregulated in the *nm2* mutant, with the fold-changes of 2.37 and 86.16, respectively. These results were consistent with previous studies, which indicated that the DGE data were accurate and reliable.

The results of qRT-PCR for 29 genes among 39 total genes were completely consistent with the DGE results. The results for 6 of the remaining 10 genes were completely inconsistent with the DGE results. The remaining 4 genes did not show a significant difference between the DGE and qRT-PCR results, which are indicated with # in Table 2. Thus, 33 genes were consistent between the DGE and qRT-PCR analyses, and the accuracy rate reached 84.62%, demonstrating that the DGE results were reliable.

## Discussion

### Differentially expressed genes based on DGE analysis

A large number of biosynthesis and decomposition processes occur during molting, such as carbohydrate and lipid metabolism, protein metabolism and nucleic acid metabolism [22, 23], and many substances are decomposed in apolysis, while many substances are synthesized in the formation of a new epidermis [24, 25]. Because of the non-molting phenotype of the *nm2* mutant, the synthesis and metabolism of proteins, lipids, glucose and nucleic acids are greatly altered. We verified 15 genes selected from the DGE results using qRT-PCR, 12 of which were consistent with the DGE analysis. Four of these genes encode glycoside hydrolase; 2 encode cuticular proteins; 2 encode nuclear receptors; 3 encode glucose-methanol-choline oxidoreductase; and one encodes peptidase.

*Hydrolase* is a gene superfamily including protein hydrolase, lipid hydrolase, glycoside hydrolase and nuclear acid hydrolase [26–28]. Because *nm2* cannot molt in the pre-molting stage of the 2^nd^ instar, the expression of hydrolases differed between the *nm2* mutant and the wildtype. The epidermis is one of the most important and largest tissues in insects, with the main function of protecting the insect from danger. The CPGs are a large family, with more than 1% of the genes in the insect genome encoding cuticular proteins [7]. Because the main phenotype of the *nm2* mutant is that it cannot molt in the pre-molting stage of the 2^nd^ instar, many CPGs might be differentially expressed in this mutant. Glucose is a molecule of prime importance, functioning as an energy substance, as a substrate for synthesizing other substances, as a component of glycoproteins and in recognition processes [29–31]. Glucose-methanol-choline oxidoreductase (GMC oxidoreductase) is homologous to *D*. *melanogaster* glucose dehydrogenase, *E*. *coli* choline dehydrogenase, *A*. *niger* glucose oxidase, *H*. *polymorpha* methanol oxidase and *B*. *sterolicum* cholesterol oxidase, which all contain a canonical ADP-binding beta alpha beta-fold close to their amino termini [32, 33]. Additionally, Chitin can bind cuticular proteins to form the cuticle, and chitin is synthesized from glucose [29]. In the *nm2* mutant, the ecdysone titer was lower than in the wildtype, and the mutant could be rescued by feeding with cholesterol. The lack of cholesterol might be related to cholesterol oxidase, which belongs to the GMC oxidoreductase family [33]. Chitin can combine with cuticular proteins to participate in the formation of the epidermis, and the *nm2* mutant accordingly cannot molt. In addition, the *nm2* mutant ingests smaller amounts of mulberry leaves and shows almost no development [15], which might be related to abnormalities in glucose metabolism.

### Differentially expressed genes in the juvenile hormone synthesis pathway

**Fig 6:**
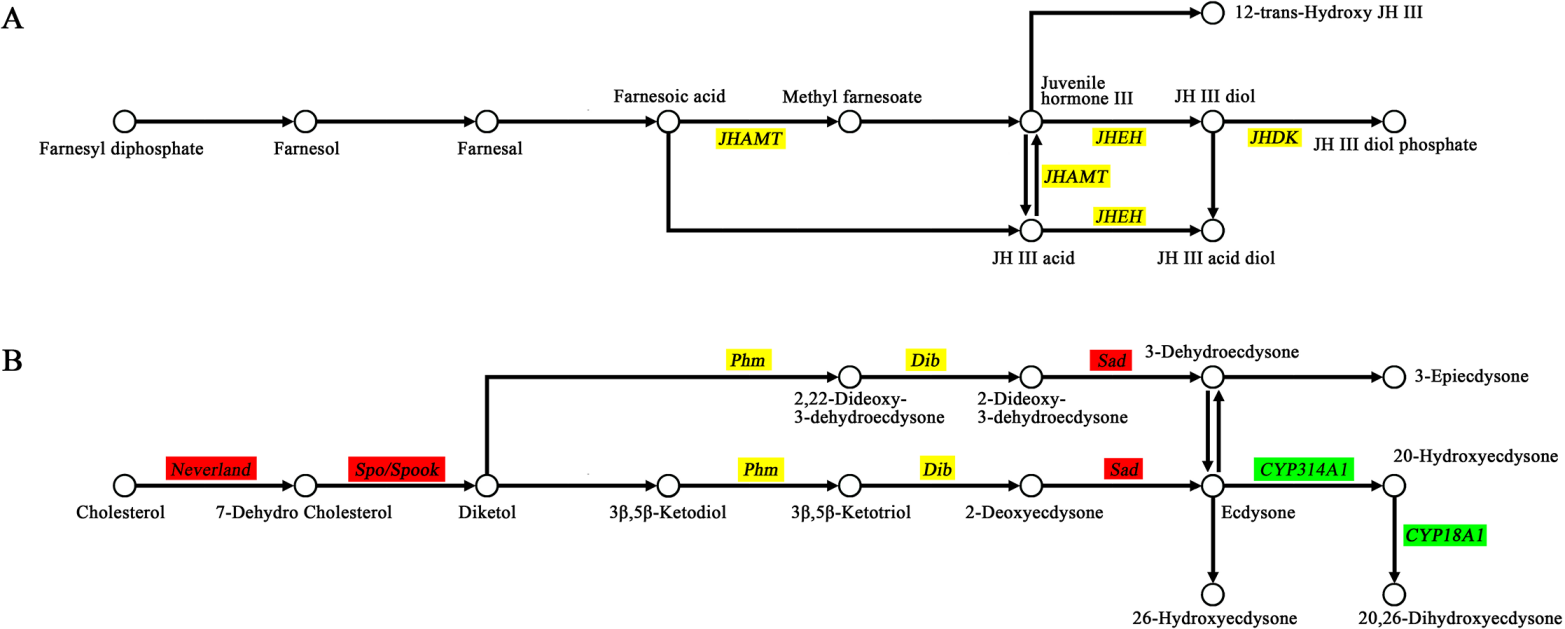
Synthesis pathways of juvenile hormone ^A^ and ecdysone ^B^. Genes with green background represent their downregulation and red for upregulation in *nm2* mutant, and yellow background represent no significant differences between C603 and *nm2*. Three key genes in the juvenile hormone biosynthesis pathway, *JHDH*, *JHDK* and *JHAMT*, were without any significant difference in the *nm2* mutant. In the ecdysone synthesis pathway, *neverland*, *spook* and *sad* were upregulated in the *nm2* mutant, and *CYP314A1* and *CYP18A1* were downregulated. There were no significant difference for *Phm* and *Dib*.

Juvenile hormone, a physiologically active substance produced in the corpora allata, has a variety of natural active forms. It plays roles in many physiological process, such as the maintenance of larval morphological traits, promotion of gonad maturity, adult diapause and pheromone production [34, 35]. Juvenile hormone always works together with ecdysone to regulate insect growth and development. The results of the DGE and qRT-PCR analyses showed no obviously differentially expressed genes in the juvenile hormone biosynthesis pathway between the wildtype and the *nm2* mutant. The key genes JHDH and JHDK were only slightly downregulated, without any significant difference (Fig 6A). Based on the above results, we speculated that the biosynthesis of juvenile hormone was not altered in the *nm2* mutant. The content of juvenile hormone also did not present a significant difference, which could be preliminarily verified by the finding that the characteristics of the *nm2* mutant exhibited no improvement after feeding with juvenile hormone (data not shown).

### Differentially expressed genes in the ecdysone synthesis pathway

Ecdysone is one of the most important hormones in metamorphosis during insect development. During the larval stages, ecdysone promotes the molting process in every stage, together with juvenile hormone. In the metamorphosis stage, ecdysone individually promotes metamorphosis [36]. The synthesis of ecdysone from the substrate cholesterol involves a series of halloween genes, and ecdysone then functions as the activated form 20-hydroxyecdysone (20E) [37]. It has been demonstrated that the 20E titer in the *nm2* mutant is significantly lower than in the wildtype. Additionally, the mutant characteristics can be rescued by feeding with 20E, cholesterol or 7-dehydrocholesterol (7dC) [15]. These results confirmed the lack of 20E in the *nm2* mutant, which might be caused by a lack of cholesterol.

In the ecdysone synthesis pathway, *neverland*, *spook* and *sad* were found to be upregulated in the *nm2* mutant and *CYP314A1* and *CYP18A1* to be downregulated (Fig 6B). Because of the lack of 20E in the *nm2* mutant, early genes such as *neverland* (fold = 11.64) and *spook* (fold = 2.84) were observed to be upregulated in an attempt to increase the production of 20E, which is controlled by feedback regulation. Other genes in the ecdysone biosynthesis pathway also exhibited a slight increase expression, with the *sad* gene being particularly upregulated (5.47-fold). However, the upregulation of these genes could not rescue the absence of 20E because of the lack of cholesterol. Thus, very little ecdysone was transformed to 20E, and interestingly, we found that the *CYP314A1* gene (fold = 0.05), which plays a role in the conversion from ecdysone to 20E [37, 38], was markedly downregulated in the *nm2* mutant. The *CYP18A1* gene was also downregulated (0.43-fold) because of the lack of 20E (shown in Table 2 and Fig 6B).

### Differentially expressed genes in the chitin synthesis pathway

Chitin, whose main component is glycosaminoglycan [39], forms the cuticle of the insect epidermis, trachea and peritrophic membrane of digestive tube, by binding proteins to protect the insect from damage [40]. The synthesis of chitin begins with glucose, which is ultimately converted to UDP-N-acetylglucosamine [29]. Chitin synthesis is a complex process, and many enzymes function in this pathway [41]. We chose three key enzymes for analysis: glucose-6-phosphate isomerase (*G6PI*), glutamine: fructose-6-phosphate-aminotransferase (*GFPA*) and UDP-N-acetylglucosamine pyrophosphorylase (*UNAP*), of which UNAP is the rate-limiting enzyme [41]. In the chitin metabolism pathway, after chitin is degraded to oligomers via chitinase, β-N-acetyl-glucosaminidase (*NAG*) converts the oligomers to monomers [42]. The results of DGE and qRT-PCR showed no obviously differences between *nm2* and the wildtype, which indicated that the synthesis of chitin occurred normally and that the content of chitin might remain at a normal level in the *nm2* mutant. Chitin can bind cuticular proteins to participate in epidermis construction following the appropriate signal, which is consistent with the finding that the *nm2* mutant can be rescued by feeding 20E.

### Differentially expressed cuticle protein genes

**Fig 7:**
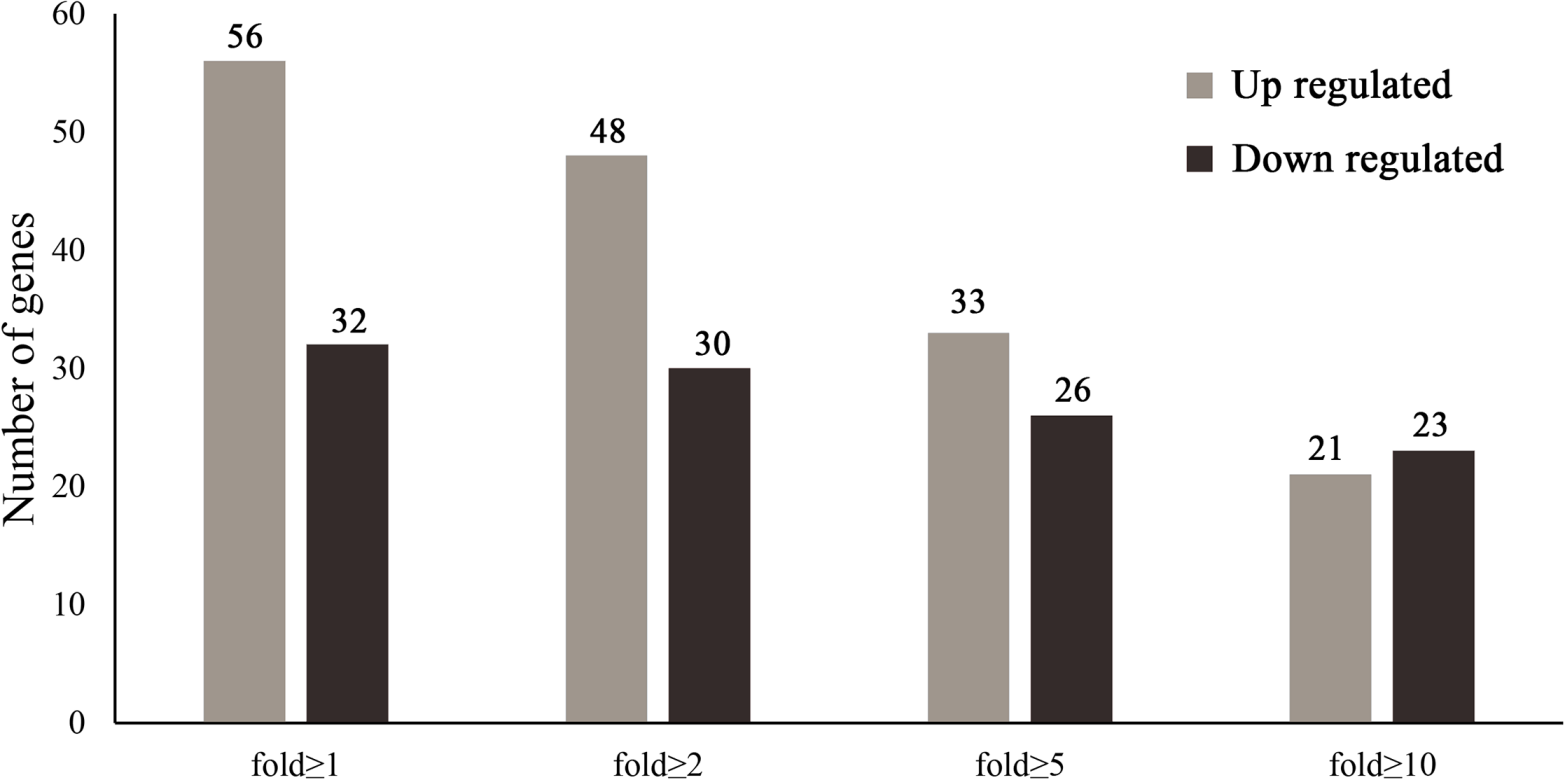
Expression of cuticle protein genes. Many *CPG*s were expressed differentially between *nm2* and C603, with more than 20 differentially expressed genes showing a fold-change of 10 or greater.

The epidermis is an important insect organ that can protect the insect from harm. The epidermis is prerequisite for growth, reproduction and adaptation to the complex and changeable living environment experienced by insects [7, 43]. In the insect genome, there are many *CPG*s, encoding a variety of cuticular proteins, and there are at least 200 *CPG*s in the silkworm [7, 8]. The analysis of 208 *CPG*s between *nm2* and the wildtype showed that 59 *CPG*s were not expressed in the wildtype and *nm2*; 81 *CPG*s were upregulated in *nm2*; and 68 *CPG*s downregulated (S5 Table). After removing 61 genes exhibiting low expression, with an FPKM<5, in both the wildtype and *nm2*, 56 genes were upregulated, and 32 genes were downregulated in *nm2*. Considering genes showing a fold-change ≥2, ≥5 or ≥10 led to the identification of 48, 33 and 21 upregulated genes and 30, 26 and 23 downregulated genes in *nm2*, respectively (see Fig 7). Based on the *CPG* statistics, we identified many *CPG*s that were expressed differentially between the wildtype and *nm2*, including many genes exhibiting very large differences. Many *CPG*s have ecdysone binding sites, and their expression is regulated by the ecdysone titer [44–47]. The ecdysone titer was lower in the *nm2* mutant than in the wildtype, and *CPG*s controlled by ecdysone were therefore expressed differentially.

### Differentially expressed ecdysone-induced nuclear receptor genes

The nuclear receptor gene superfamily consists of transcription factors that participate in many biological processes, including molting metamorphosis, embryonic development, cell differentiation, reproduction and other physiological processes [9, 48], and many nuclear receptor genes are involved in the ecdysone-induced signaling pathway. Ashburner [49] first established this signaling pathway in 1973; after verification by a number of scholars, Bonneton et al. [50] amend the pathway in 2008. In this pathway, early genes such as *E75*, *Broad*, *E74*, *E93* and *HR39* are first activated after the binding of 20E and EcR-Usp heterologous dimers, followed by the activation of early-late genes such as *HR3*, *HR4*, *HR38* and *E78* and, finally, the activation of late genes, by amplifying the activation signal through βFTZ-F1. By analyzing silkworm nuclear receptor genes [51], we found that the key gene *βFTZ-F1* was significantly upregulated (5.04-fold); the early-late genes *HR3* and *HR4* were significantly downregulated; and no significant difference was observed for other nuclear receptor genes, with usp being slightly upregulated, whereas the others were slightly downregulated (Table 2).

*βFTZ-F1* is expressed in late embryonic development and in the larval and pupal stages at each developmental transition in insects and is controlled by 20E. *βFTZ-F1* expression significantly increases when the 20E titer is low [52]. In addition, *βFTZ-F1* promotes ecdysone biosynthesis during molting [53]. In the *nm2* mutant, the 20E titer is much lower than in the wildtype, and the mutant can be rescued by feeding with 20E. A low 20E titer causes an increase in βFTZ-F1, which is consistent with the significant upregulation of *βFTZ-F1* observed in the *nm2* mutant. The functions of *HR3* and *HR4*, which are early-late genes in the ecdysone-induced signaling pathway, are similar in the activation of relatively late genes. *HR3* is closely related to insect molting [54], and *HR4* is involved in ovum formation [55]. The *nm2* mutant is non-molting in the 2^nd^ instar, which might be related to downstream genes regulated by *HR3* and *HR4*. The DGE results showed many *CPG*s were differentially expressed between the wildtype and *nm2*, and these *CPG*s might be controlled by *HR3* and *HR4* and be differentially expressed along with the downregulation of *HR3* and *HR4*.

### BmCPG10 is upregulated in the *nm2* mutant

An approximately 217 bp deletion in the open reading frame (ORF) of the *BmCPG10* gene was found to be the key cause of the *nm2* mutant phenotype. The results of a semi-quantitative reverse-transcription polymerase chain reaction (RT-PCR) analysis showed *BmCPG10* to be highly expressed in the wildtype C603 and not expressed in the *nm2* mutant [15]. However, our DGE results indicated that *BmCPG10* was upregulated in the *nm2* mutant (see Table 2). After analyzing *BmCPG10* in the *nm2* mutant, we found that the RT-PCR primers targeted the mutant region where the 217 bp deleted sequence was located, which resulted in the false observation that *BmCPG10* was not expressed in the *nm2* mutant because the 217 bp deleted sequence did not exist in the *nm2* mutant, leading to the absence of an RT-PCR product. Hence, we used a pair of primers targeting the normal region of the gene for qRT-PCR, and the results showed that *BmCPG10* was indeed upregulated in the *nm2* mutant (Table 2).

### Possible formation mechanism of the *nm2* mutant

According to previous research, cuticular proteins are thought to be regulated by the 20E titer, as late genes in the ecdysone-induced signaling pathway [56]. When the 20E titer reaches a threshold value, 20E combines with the ecdysone receptor and promotes a series of genes, including a series of *CPG*s, to complete the molting process [57, 58]. A large number of genes in the ecdysone-induced signaling pathway were expressed abnormally because of the low titer of 20E in the *nm2* mutant. The *nobo* gene is essential for ecdysteroid biosynthesis via regulating the behavior of cholesterol [59], and this gene was found to be slightly upregulated. Most genes in the ecdysone synthesis pathway were upregulated, while the *CYP314A1* gene, catalyzing the transformation from ecdysone to 20E, was markedly downregulated. The nuclear receptor genes in the ecdysone-induced signaling pathway were also expressed differentially, with most of these receptor genes being downregulated, except for *FTZ-F1* and *USP*.

**Fig 8:**
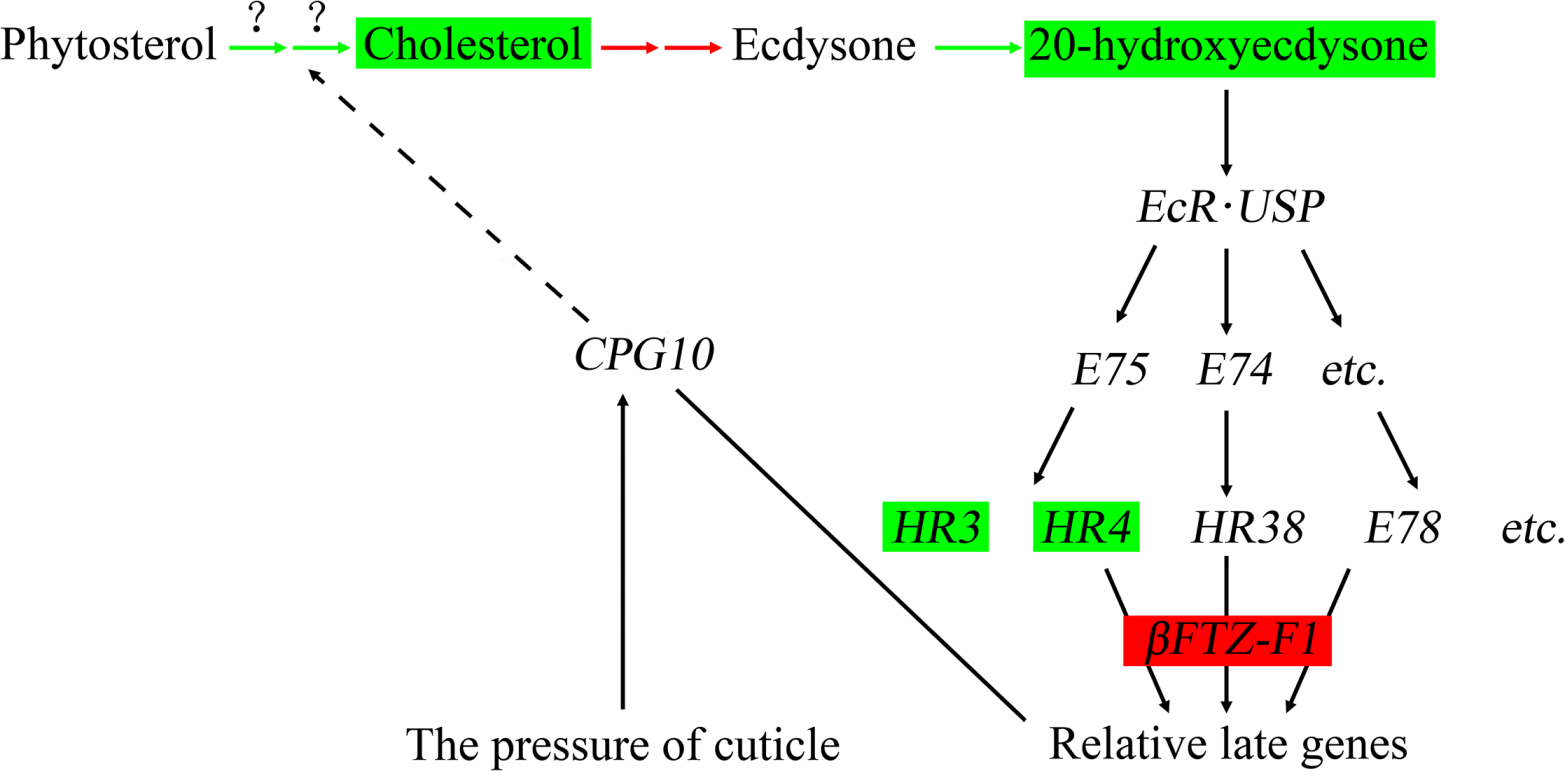
The ecdysone-induced signaling pathway. The 20E titer in the *nm2* mutant was lower than in the wildtype, leading to upregulation of ecdysone biosynthesis genes and downregulation of *CYP314A1*, which takes part in the conversion from ecdysone to 20E. Two nuclear receptor genes, *HR3* and *HR4*, were markedly downregulated, whereas the key nuclear receptor gene *βFTZ-F1* was significantly upregulated. *BmCPG10* might act as an EPDFP monitor to regulate the molting process by controlling the biosynthesis of ecdysone.

The previous research confirmed that the cuticular protein gene *BmCPG10* is responsible for the phenotype of the *nm2* silkworm mutant. The 20E titer in *nm2* is lower than that in the wildtype, and the *nm2* mutant can be rescued by feeding with 20E [15], indicating that the 20E titer is regulated by *BmCPG10* to some extent. However, *CPG*s always act as effectors in the ecdysone-induced signaling pathway [2, 56]. Based on these findings, we conjecture that *BmCPG10* might be endowed with new functions. A possible mechanism underlying the development of the *nm2* mutant phenotype is suggested in Fig 8.

The cuticle of insects does not exhibit elasticity and cannot grow with the growth and development of larvae. Therefore, molting must occur every time the cuticle is no longer sufficiently large to accommodate the growth and development of larvae [60]. In the molting process, molting hormone and juvenile hormone play key roles. Additionally, illumination, temperature, nutrition and the expansion pressure and distraction forces of polypides (EPDFP) can promote the process of molting [61, 62]. However, the possible mechanism promoting molting is still unclear. We speculate that *BmCPG10* is one type of molting promoter that may monitor EPDFP. When EPDFP occurs at a low level, *BmCPG10* is highly expressed, but when EPDFP reaches a threshold value, the expression of *BmCPG10* declines to a low level; during this process, the molting process is promoted. Furthermore, *BmCPG10* is necessary for cholesterol generation via ecdysteroid synthesis or ecdysteroid transportation. In the *nm2* mutant, the function of *BmCPG10* is lost, and it cannot play the role monitoring EPDFP. Hence, when the silkworm larvae meet the requirements for molting in the 2^nd^ instar, *BmCPG10* cannot respond to EPDFP, and the expression of *BmCPG10* remains at a high level (3.56-fold); thus, the molting process is not promoted. In the 1^st^ instar, another CPG might act as the EPDFP monitor to promote the first molt, explaining why the phenotype of non-molting in the 2^nd^ instar occurs in the *nm2* mutant.

## Conclusion

Several ecdysone synthesis genes were found to be significantly upregulated in *nm2* as a result of a lack of 20E, while *CYP314A1* is significantly downregulated in *nm2* due to a lack of ecdysone. As the effectors of the ecdysone-induced signaling pathway, many CPGs, including the mutated *BmCPG10* gene of the *nm2* mutant, are expressed differentially between the wildtype and *nm2*. Three ecdysone-induced nuclear receptor genes (*FTZ-F1*, *HR3* and *HR4*) are greatly affected by the lack of 20E.

## Supporting information

S1 Table Primers for qRT-PCR, functional description and qRT-PCR and FPKM results. (XLSX)

S2 Table FPKMs of all genes in *nm2* and C603. (XLSX)

S3 Table Differentially expressed genes between *nm2* and C603. (XLSX)

S4 Table GO enrichment and KEGG enrichment analyses of differentially expressed genes. (XLSX)

S5 Table Expression of cuticular protein genes. (XLSX)
